# Escalating Catalytic Activity for Hydrogen Evolution Reaction on MoSe_2_@Graphene Functionalization

**DOI:** 10.3390/nano13142139

**Published:** 2023-07-23

**Authors:** Hoa Thi Bui, Nguyen Duc Lam, Do Chi Linh, Nguyen Thi Mai, HyungIl Chang, Sung-Hwan Han, Vu Thi Kim Oanh, Anh Tuan Pham, Supriya A. Patil, Nguyen Thanh Tung, Nabeen K. Shrestha

**Affiliations:** 1 Institute of Materials Science, Vietnam Academy of Science and Technology, Hanoi 100000, Vietnam; lamnd@ims.vast.ac.vn (N.D.L.); linhdc@ims.vast.ac.vn (D.C.L.); maint@ims.vast.ac.vn (N.T.M.); 2 Department of Chemistry, Hanyang University, 222, Wangsimni-ro, Seongdong-gu, Seoul 04763, Republic of Korea; doctor99106@gmail.com (H.C.); shhan@hanyang.ac.kr (S.-H.H.); 3 Institute of Physic and Graduate University of Science and Technology, Vietnam Academy of Science and Technology, Hanoi 100000, Vietnam; oanhvtk@iop.vast.vn; 4 Institute of Engineering and Technology, Thu Dau Mot University, Binh Duong 75000, Vietnam; anhpt195@tdmu.edu.vn; 5 Department of Nanotechnology and Advanced Materials Engineering, Sejong University, Seoul 05006, Republic of Korea; supriya2812@sejong.ac.kr; 6 Division of Physics and Semiconductor Science, Dongguk University, Seoul 04620, Republic of Korea

**Keywords:** MoSe_2_@Gr, graphene incorporation, hydrogen evolution reaction

## Abstract

Developing highly efficient and durable hydrogen evolution reaction (HER) electrocatalysts is crucial for addressing the energy and environmental challenges. Among the 2D-layered chalcogenides, MoSe_2_ possesses superior features for HER catalysis. The *van der Waals* attractions and high surface energy, however, stack the MoSe_2_ layers, resulting in a loss of edge active catalytic sites. In addition, MoSe_2_ suffers from low intrinsic conductivity and weak electrical contact with active sites. To overcome the issues, this work presents a novel approach, wherein the in situ incorporated diethylene glycol solvent into the interlayers of MoSe_2_ during synthesis when treated thermally in an inert atmosphere at 600 °C transformed into graphene (Gr). This widened the interlayer spacing of MoSe_2_, thereby exposing more HER active edge sites with high conductivity offered by the incorporated Gr. The resulting MoSe_2_-Gr composite exhibited a significantly enhanced HER catalytic activity compared to the pristine MoSe_2_ in an acidic medium and demonstrated a superior HER catalytic activity compared to the state-of-the-art Pt/C catalyst, particularly at a high current density beyond ca. 55 mA cm^−2^. Additionally, the MoSe_2_-Gr catalyst demonstrated long-term electrochemical stability during HER. This work, thus, presents a facile and novel approach for obtaining an efficient MoSe_2_ electrocatalyst applicable in green hydrogen production.

## 1. Introduction

Fossil fuels are the major contributor to the energy landscape, but their depletion and negative environmental impacts have prompted a search for alternative energy solutions [1,2,3,4]. Toward this perspective, various renewable energies have been studied and developed. Among them, hydrogen has been studied extensively and is considered a promising energy carrier due to its clean combustion and high energy density [5,6,7,8,9,10,11]. To date, steam methane reforming is a widely employed route for the industrial-scale production of hydrogen. In addition to the high energy supply requirement for maintaining high temperature and pressure in the reforming process, this route also produces carbon mono- and di-oxides as byproducts. Therefore, this conventional method of hydrogen production needs to substitute urgently with a green technique that runs with zero carbon emission and does not require a high energy supply. Electrochemical water slitting is a green route to obtain molecular oxygen and hydrogen. Hence, to meet global energy demands and address environmental concerns, producing hydrogen through water splitting has recently gained increasing interest [12,13]. Platinum-based catalysts are currently the most effective for the hydrogen evolution reaction (HER), exhibiting nearly zero onset overpotential and a low Tafel slope [14,15]. However, their limited availability and high cost hindered widespread applicability. Consequently, considerable work has been carried out to develop HER electrocatalysts that are equally efficient and durable but more affordable [16,17,18,19,20,21,22].

Transition metal dichalcogenides (TMDs) such as sulfide, selenide, and telluride of molybdenum and tungsten with layered structures have been paid considerable attention in various fields, including catalysts, energy storage, transistors, and photoelectrochemical devices [23,24,25,26,27,28,29]. The unique two-dimensional (2D) architecture with excellent electrical properties and good catalytic activity offers TMDs as promising candidates for HER electrocatalysts [30,31,32]. Among them, MoS_2_ has been studied extensively and employed as an HER catalyst [33,34]. In the same group of TMDs, MoSe_2_ bears similar layer-to-layer structures and exhibits versatile electrochemical properties with good stability. MoSe_2_ possessed a metallic nature, offering superior electrical conductivity compared to MoS_2_, which is beneficial for HER catalysts [35,36]. However, the high surface energy and *van der Waals* attractions of MoSe_2_ interlayers lead to the stacking of the layers, resulting in a loss of active catalytic sites [37,38]. Additionally, MoSe_2_ suffered from low intrinsic conductivity and weak electrical contact with active sites, which negatively impacts its catalytic activity. To overcome these challenges, researchers are actively exploring various approaches to improve the catalytic performance of MoSe_2_. These approaches include the manipulation of nanostructures to improve conductivity and electron transfer, optimizing the composition and surface modification, and exploring hybrid structures with conductive carbon materials [39,40,41,42,43].

Herein, this work demonstrates the successful synthesis of the MoSe_2_-Gr composite via a two-step process involving solvothermal synthesis of MoSe_2_ using diethylene glycol solvent followed by thermal treatment to create MoSe_2_-Gr. The resulting MoSe_2_-Gr catalyst exhibited highly competitive electrocatalytic performance in acidic media for the HER. Specifically, this catalyst exhibited lower overpotentials of 161 and 250 mV at a current density of 10 and 50 mA.cm^−2^, respectively, and a small Tafel slope of 67 mV dec^−1^. It is worth noting that this performance lies on the top tire of MoSe_2_ and their carbonous composites based HER electrocatalysts synthesized by various approaches (see, Table S1 in the Supporting Information Section). Most importantly, it should be further noted that the MoSe_2_-Gr catalyst designed in this work demonstrated a superior HER catalytic activity than that of the state-of-the-art Pt/C catalyst, especially when the electrolysis was performed at a high cathodic current density beyond ca. −55 mA cm^−2^. Such catalysts exhibiting superior HER catalytic activity at high current density are particularly applicable for the industrial scale of hydrogen production. The observed superior HER performance of the MoSe_2_-Gr catalyst can be owing to the amplified surface area and concentration of active edge sites present within the wider interlayer width of MoSe_2_. Moreover, the resulting architecture of the composite facilitated the efficient movement of charges and ions between the MoSe_2_ interlayers and electrolytes, thereby enhancing the charge and mass transfer capability. Additionally, the MoSe_2_-Gr catalyst exhibited prolonged electrochemical stability in acidic electrolytes during the HER.

## 2. Materials and Methods

### 2.1. Regents and Materials

Red phosphorus (Sigma-Aldrich), selenium powder (Sigma-Aldrich), sodium molybdenum oxide dihydrate (Na_2_MoO_4_·2H_2_O, Sigma-Aldrich), Sodium sulfate (Na_2_SO_4_); Nafion (TM perfluorinated resin solution, Sigma-Aldrich), sulfuric acid (H_2_SO_4_), diethylene glycol (HOCH_2_CH_2_)_2_O: DEG, Sigma-Aldrich) and benzene (C_6_H_6_, Scharlau) were used in their as-received state without undergoing additional purification. For preparing the reagent solutions, deionized water (DI: H_2_O) was used throughout the experiments.

### 2.2. Preparation of MoSe_2_ and MoSe_2_-Graphene (MoSe_2_-Gr)

#### 2.2.1. Preparation of Selenoacetamide

A 2:5 mole ratio of red phosphorus and selenium powder mixture in a test tube was flushed with argon gas for 15 min before being heated by a burner flame until completed to form P_2_Se_5_. Then, a suspension of synthesized powder P_2_Se_5_ in acetonitrile was prepared in a round bottom flask and refluxed at 80 °C for 6 h., DI H_2_O was added dropwise during refluxing to neutralize the suspension. After cooling down, it was filtered and extracted by benzene. After being dried by Na_2_SO_4_, filtered, and condensed, the selenoacetamide powder was obtained.

#### 2.2.2. Synthesis of MoSe_2_ and MoSe_2_-Graphene (MoSe_2_-Gr)

For the synthesis of selenides, a mixture of 5 mmol Na_2_MoO_4_·2H_2_O and 20 mmol selenoacetamide was first dissolved in 40 mL of diethylene glycol (DEG) by stirring for 30 min. The mixture solution was transferred into a 50 mL capacity Teflon-lined autoclave. After sealing the autoclave carefully, it was heated at 200 °C for 15 h. After the completion of the reaction, the reflux system was cooled down to room temperature, and the products were centrifuged thrice for 30 min each with DI H_2_O and ethanol. The collected MoSe_2_ powder was dried for at least 24 h in a vacuum oven at 80 °C.

Transformation of MoSe_2_ into MoSe_2_-Graphene (MoSe_2_-Gr) composite was conducted by calcinating the synthesized MoSe_2_ powder with a constant supply of N_2_ (99.9999%). For this, the MoSe_2_ powder was placed in a tube furnace, and the temperature was ramped at a rate of 5 °C min^−1^ until the final temperature reached 600 °C. The annealing continued for 5 h and the system was cooled down naturally to room temperature.

### 2.3. Characterizations

Surface topography of the materials was obtained by field emission scanning electron spectroscopy (FE-SEM, HITACHI S-4800) operated at an accelerating voltage of 15 kV. The crystal structure of the synthesized materials was investigated via X-ray diffraction analysis (XRD; Rigaku D/MAX 2600 V, Cu Kα (α = 0.15418 nm)). High-resolution transmission electron microscopy (HR-TEM) and selected area electron diffraction (SAED) analysis techniques were employed to study the crystal properties of the materials. The HR-TEM analysis was conducted using a Jeol JEM2100 instrument at an acceleration voltage of 200 kV. The carbon-supported grids were used for the sample preparation of TEM. The chemical composition was examined via an energy-dispersive X-ray analyzer (EDX), which was integrated into the TEM. Scanning transmission electron microscopy (STEM) was employed to perform elemental mapping of the materials. Raman shifts of materials were recorded via a Renishaw in via Raman system operating at an exciting wavelength of 532 nm. Additionally, the elements (C, H, and N) analysis was performed via an elemental analyzer (FLASH EA1112). The surface area and pore volume of the material were determined using the Brunauer–Emmett–Teller (BET) method, with nitrogen adsorption/desorption measurements (Micromeritics ASAP 2060).

#### Electrochemical Characterization and HER Activity Measurement

A glassy carbon electrode (GCE, diameter = 3 mm) was used as the support for depositing the catalyst films. For the film formation, catalyst ink was prepared by dispersing finely grounded 5 mg of catalysts in 1 mL of DI H_2_O: IPA: Nafion mixture with a volume ratio of 4.5:5:0.5, respectively. The mixture was sonicated and stirred continuously until it became homogenous ink. The ink was drop-casted carefully onto the GCE surface and dried for at least 24 h at room temperature prior to use. The catalyst loading on the GCE was 0.25 mg.cm^−^^2^.

The electrochemical measurements were carried out using the COMPACTSTAT Electrochemical Interface and Impedance Analyzer (IVIUM Technologies) in a standard three-electrode system. The counter electrode consisted of a graphite rod, the reference electrode was Ag/AgCl (3 M NaCl), and the working electrode was the GCE with the catalyst drop-casted onto it. For the electrolysis, an aqueous solution of 0.5 M H_2_SO_4_ was used as electrolyte, which was de-aerated for 30 min by bubbling with Argon gas prior to the measurements.

The electrocatalytic performance of the catalyst on HER was accessed by using linear sweep voltammetry in an acidic electrolyte (0.5 M H_2_SO_4_) at a scan rate of 5 mV s^−1^ in the potential range from +0.1 to −0.80 V vs. RHE (reversible hydrogen electrode) without an *iR* compensation. The bias potential (E_Ag/AgCl_) applied against the Ag/AgCl reference electrode was converted into the potential on the RHE scale using the equation E (RHE) = E_Ag/AgCl_ + 0.059pH + E^0^_Ag/AgCl_, where E^0^_Ag/AgCl =_ 0.21 V at 25 °C [44,45]. To evaluate the durability of the catalyst for HER, a chronopotentiometry measurement was performed in 0.5 M H_2_SO_4_ aqueous electrolytes at a cathodic bias of −10 mA.cm^−2^. Moreover, for more understanding of the electrochemical kinetics in HER, the electrochemical impedance spectroscopy (EIS) was measured at an external bias of −0.175 V vs. RHE in a frequency range of 100,000 Hz to 0.01 Hz.

Additionally, the mass activity = *j*/*m* relationship was used to calculate the mass activity of an electrocatalyst [46], in which *j* is the measured geometrical area-based current density (mA cm^−2^) and *m* is the mass loading of the catalyst (mg cm^−2^).

## 3. Results and Discussions

**Figure 1:**
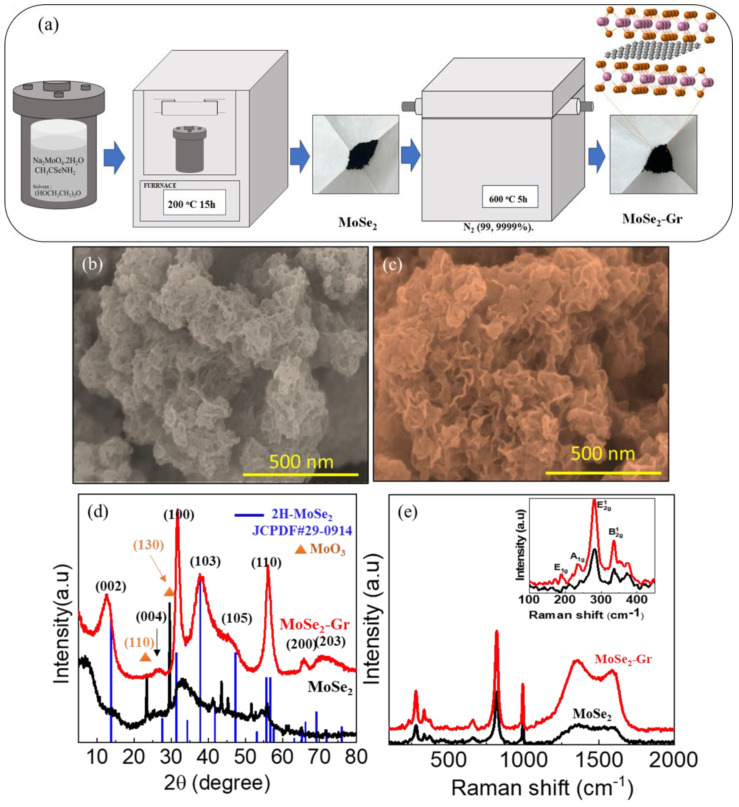
(**a**) Schematic diagram displaying the synthesis process of MoSe_2_ and MoSe_2_-Gr. SEM images of (**b**) MoSe_2_ and (**c**) MoSe_2_-Gr; (**d**) XRD patterns, and (**e**) Raman spectra of MoSe_2_ and MoSe_2_-Gr (inset image shows Raman spectra of MoSe_2_ (black color) and MoSe_2_-Gr (red color) in the Raman shift of 100~450 cm^−1^).

Figure 1a depicts the schematic diagram for the synthesis of pristine MoSe_2_ and MoSe_2_-Gr samples, which involved the solvothermal reaction of Mo and Se precursor in DEG solvent at 200 °C for 15 h to produce MoSe_2_, followed by thermal treatment in an inert atmosphere at 600 °C for 5 h to form MoSe_2_-Gr.

The SEM surface topography of the obtained MoSe_2_ and MoSe_2_-Gr samples is shown in Figure 1b,c, respectively. Compared to the as-synthesized MoSe_2_, the surface morphology of the MoSe_2_-Gr after the calcination looks significantly different. The resulting MoSe_2_-Gr nanosheets assembled to a porous architecture are clearly observable, indicating an enhanced surface area. This feature contributes to higher catalytic active sites with facile movements of ions and charges between the MoSe_2_-Gr catalyst and electrolyte, thereby significantly improving the electrocatalyst performance. To gain further insight into the surface area and pore size distribution of MoSe_2_ and MoSe_2_-Gr, BET nitrogen adsorption/desorption isotherm measurements were conducted and are presented in Figure S1. Figure S1a,b show the nitrogen sorption isotherms of the pristine MoSe_2_ and MoSe_2_-Gr, respectively, both exhibiting type IV isotherms. The specific surface areas of MoSe_2_ and MoSe_2_-Gr were determined to be 13.15 m^2^ g^−1^ and 38.33 m^2^ g^−1^, respectively. The significantly higher specific surface area of MoSe_2_-Gr compared to pristine MoSe_2_ is attributed to the in-situ incorporation of graphene into the MoSe_2_ interlayers. The pore size distribution of MoSe_2_ and MoSe_2_-Gr are displayed in Figure S1c,d, respectively. The pore size distribution curves indicate the presence of mesoporous structures. These observations are in agreement with the SEM analysis.

Figure 1d illustrates the XRD patterns of MoSe_2_ and MoSe_2_-Gr, and the reference 2H-MoSe_2_ (JCPDS #29-0914) has also been shown for comparison. The XRD patterns of the as-prepared MoSe_2_ sample indicate that the sample was primarily composed of MoSe_2_ with a small amount of MoO_3_ present. Specifically, the diffraction peaks at 2θ angles of 27.61°, 31.42°, 47.50°, and 56.00° can be observed, which correspond to the lattice planes of (004), (100), (105), and (110) of the crystalline 2H-MoSe_2_, respectively. However, the main (002) peak of the as-prepared MoSe_2_ sample, which is typically observed at 13.70° in 2H-MoSe_2_ is observed to be shifted significantly to a lower 2θ position of 8.00°. The observed shift indicates that the interlayer spacing of the MoSe_2_ sample has been expanded, which may have been caused by the incorporation of DEG solvent molecules into the MoSe_2_ layers. [28,47]. In addition, the XRD pattern of the MoSe_2_ sample presents the presence of two peaks at 23.42° and 28.70°, which correspond to the diffraction from (110) and (130) planes of MoO_3_ (JCPDS #05-0508). The presence of oxides can be due to various factors such as impurities present in the precursors, residual solvents, and moisture contamination from the surrounding during the synthesis process [28,29,48]. After the thermal treatment, the diffraction of the MoSe_2_-Gr sample exhibited intense peaks, indicating an improved crystallinity of the MoSe_2_-Gr sample. The diffraction peaks observed at 12.60°, 26.95°, 31.68°, 37.76°, 47.88°, 55.99°, 65.82°, and 70.55° can be precisely assigned to the (002), (004), (100), (103), (105), (110), (200) and (203) crystal planes of 2H-MoSe_2_ (JCPDS 29-0914). This finding confirms the presence of MoSe_2_ in the MoSe_2_-Gr sample. However, in this case, the (002) peak of the MoSe_2_-Gr sample showed a slight shift compared to 2H-MoSe_2_. This shift is attributed to the evaporation of trapped DEG and the carbonization of a small amount of DEG, leading to the formation of graphene within the MoSe_2_ layers during the calcination process. Interestingly, after the thermal treatment, the peaks corresponding to the oxides (110) and (130) disappeared, indicating a reduction of the oxides present initially in the MoSe_2_ sample. The Raman spectra of both MoSe_2_ and MoSe_2_-Gr samples were displayed in Figure 1e. The vibration modes including E_1g_ at 188.82 cm^−1^, out-of-plane A_1g_ at 237.25 cm^−1^, and in-plane modes E_12g_ and B_12g_ at 280.35 cm^−1^ and 337.40 cm^−1^, respectively, can be observed. These modes correspond to the 2H phase of MoSe_2_, confirming the presence of MoSe_2_ in both samples [49,50,51,52]. The Raman shift results agree well with the XRD patterns, confirming the successful formation of 2H-MoSe_2_. Additionally, in the Raman spectrum of the MoSe_2_-Gr sample, two broad bands were observed. The band at approximately 1352.40 cm^−1^ corresponds to the D-band, while the band at around 1599.29 cm^−1^ represents the G-band, both characteristic of graphene. The G-band rose from the bond stretching between all pairs of sp^2^ atoms in both rings and chains of graphene, indicating the presence of a well-ordered carbon lattice. On the other hand, the D-band was associated with disordered carbon defects or disorder-induced phonon scattering in graphene sheets. These defects can arise from the carbonization of trapped DEG during thermal treatment, resulting in the formation of graphene within the MoSe_2_ layers. The observation of these bands further supported the presence of graphene in the MoSe_2_-Gr sample.

**Figure 2:**
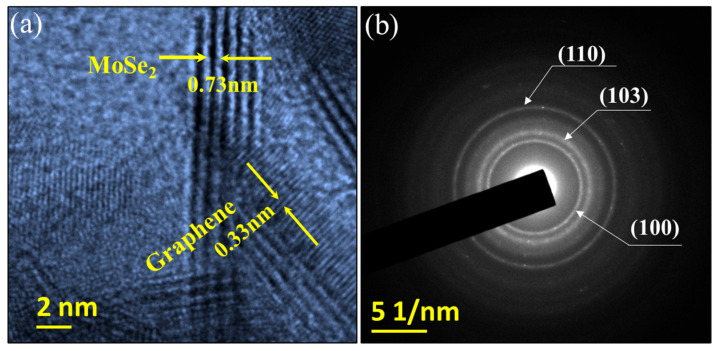
(**a**) HRTEM images and (**b**) selective area electron diffraction (SAED) patterns of MoSe_2_-Gr sample.

The crystal lattices of the MoSe_2_-Gr sample were further investigated using HR-TEM. Figure 2a displays the HR-TEM image, which reveals that the interplanar spacing of the MoSe_2_-Gr sample measures 0.73 nm, which can be attributed to the expanded interlayer distance of MoSe_2_, specifically of the (002) facet. It is important to note that the standard interplanar distance of the MoSe_2_ (002) plane is typically 0.65 nm. This implies the widening of the interlayer spacing of MoSe_2_ in the MoSe_2_-Gr sample. This broadened interplanar spacing observed in the MoSe_2_-Gr sample suggests that the *in situ* formed graphene during the calcination was incorporated within the interlayer of MoSe_2_. Furthermore, the HR-TEM image (Figure 2a) shows an additional d-spacing of 0.33 nm corresponding to the characteristic d-spacing of graphene. This observation further supports the presence of graphene within the MoSe_2_-Gr sample. Moreover, the selected area electron diffraction (SAED) patterns in Figure 2b reveal that the MoSe_2_-Gr sample has a polycrystalline structure. The diffraction rings observed in the SAED patterns correspond well with the (100), (103), and (110) planes of the hexagonal 2H-MoSe_2_ phase, which is consistent with the results obtained from the XRD analysis. Taken together, the XRD, Raman, and HR-TEM data provide conclusive evidence for the in-situ formation and incorporation of graphene within the interlayers of MoSe_2_ in the MoSe_2_-Gr sample.

**Figure 3:**
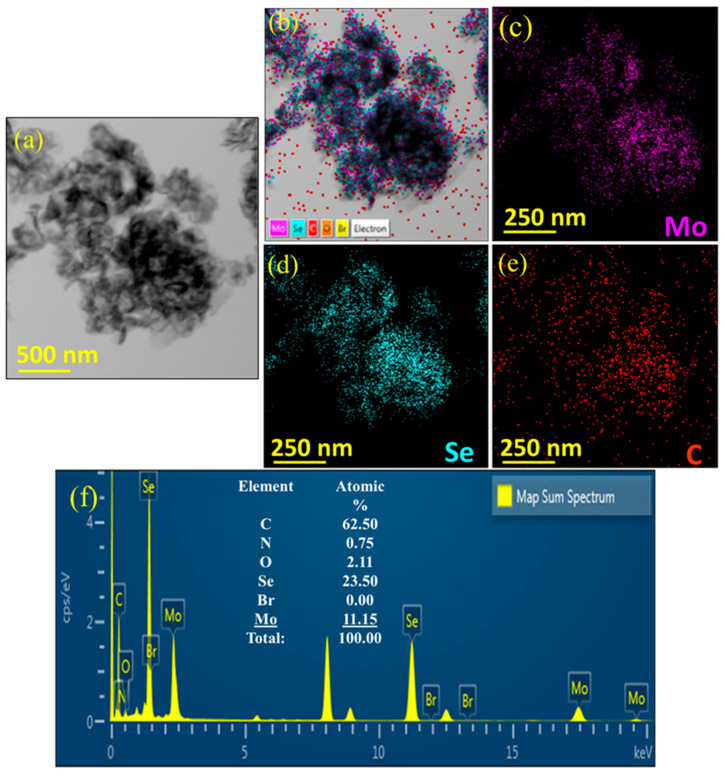
(**a**) TEM image, (**b**) HAADF-STEM image, and the corresponding TEM-EDX elemental mapping images of (**c**) Mo, (**d**) Se, and (**e**) C of the MoSe_2_-Gr sample. (**f**) EDX spectrum and the corresponding elemental atomic percentage of MoSe_2_-Gr sample.

Figure 3a–e displayed the TEM image, STEM-based high-angle annular dark-field (HAADF-STEM) image, and the corresponding TEM-based EDX elemental mapping images of Mo, Se, and C in the MoSe_2_-Gr sample. These images demonstrate a homogeneous distribution of each constituent in the MoSe_2_-Gr sample. Additionally, Figure 3f displays the EDX spectrum of the MoSe_2_-Gr sample along with the atomic percentages of each element obtained from TEM-EDX-based analysis. The atomic percentages of Mo and Se were measured to be 11.15 and 23.50%, respectively, which closely align with the expected 1:2 ratio of MoSe_2_. Since carbon-supported grids were employed for TEM characterization, the carbon content in the sample could not be measured precisely via TEM-based EDX analysis. As a result, for the analysis of carbon content, elemental analysis (N, C, and H) in the MoSe_2_ and MoSe_2_-Gr samples was conducted using an elemental analyzer (FLASH EA1112). The results are listed in Table 1. The MoSe_2_ sample exhibited a C content of 11.632 wt%. while the MoSe_2_-Gr sample showed a decrease in C content to 7.210 wt%. This can be due to the evaporation of DEG and the formation of graphene by thermal treatment, leading to a decrease in the C content, while the N content, which could be detected from contamination from the background surrounding remained relatively unchanged. Overall, the TEM-EDX analysis, along with the elemental analysis provided information on the elemental composition and distribution in the MoSe_2_-Gr sample, confirming the successful integration of graphene into the MoSe_2_ structure.

**Figure 4:**
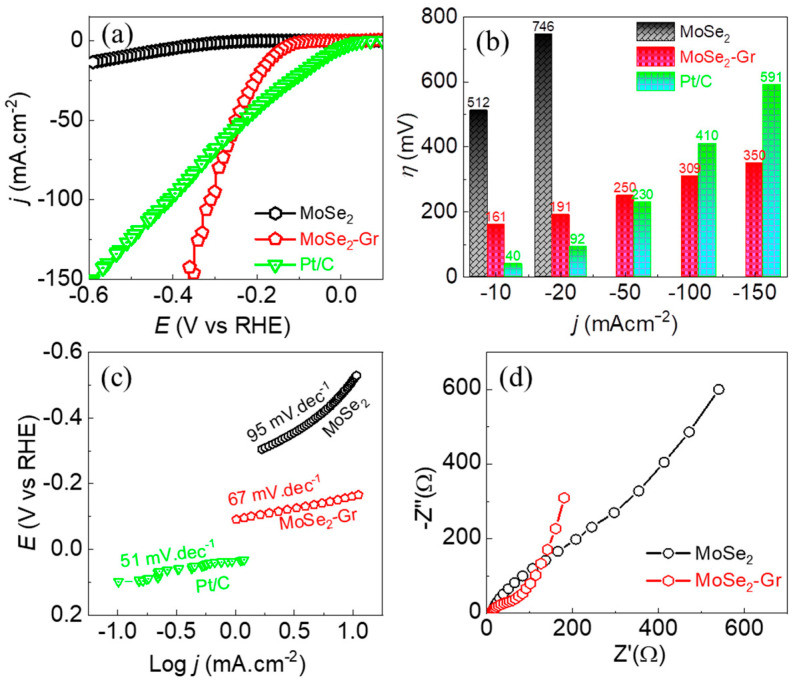
Electrochemical measurement for HER performance in 0.5 M H_2_SO_4_. (**a**) cathodic LSV curves, (**b**) corresponding HER overpotential vs. current density profile, (**c**) Tafel plots, and (**d**) electrochemical impedance spectra.

The HER performance of the MoSe_2_, MoSe_2_-Gr, and the benchmark Pt/C electrocatalysts was evaluated in a typical three-electrode electrochemical cell. The working electrode was a drop-casted catalyst film on a GEC electrode. Prior to the measurements, the working electrodes were activated and stabilized via cycling at a scanning rate of 50 mVs^−1^ in a potential window of +0.1 to −0.4 V vs. RHE until constant voltammograms were obtained. The electrocatalytic activity for the HER was accessed via linear sweep voltammograms (LSV) recorded without an iR correction. Figure 4a displays the cathodic LSV curves of MoSe_2_ and MoSe_2_-Gr in 0.5 M H_2_SO_4_ solution. In addition, a commercial 20 wt.% Pt/C, which is often employed as the state-of-the-art HER electrocatalyst was also measured as the benchmark HER catalyst. The benchmark Pt/C electrocatalyst exhibited a nearly zero onset potential, reflecting superior catalytic activity toward HER. The LSV curve of the as-synthesized MoSe_2_ sample demonstrated poorer catalytic performance, which is indicated by a high overpotential of 512 mV required to achieve an HER current density of −10 mA·cm*^−^*^2^. However, after the thermal treatment, the obtained MoSe_2_-Gr catalyst showed significantly improved HER activity compared to that of the MoSe_2_ catalyst. This improvement is evident by the considerably reduced overpotential of MoSe_2_-Gr, measuring 161 mV vs. RHE to drive an HER current density of −10 mA·cm*^−^*^2^. Interestingly, the MoSe_2_-Gr catalyst achieved an HER current density of −50 mA·cm*^−^*^2^ at an overpotential of 250 mV vs. RHE, which is closer to that of the benchmark Pt/C (230 mV) shown in Figure 4a. Figure 4b provided a detailed comparison of the HER overpotentials of MoSe_2_, MoSe_2_-Gr, and the benchmark Pt/C. This comparison highlights the superior electrocatalytic performance of MoSe_2_-Gr, exhibiting lower overpotentials compared to MoSe_2_. Most importantly, the HER catalytic activity of the MoSe_2_-Gr approached the performance of the benchmark Pt/C catalyst when the HER was performed at ca. −50 mA·cm*^−^*^2^. Furthermore, the MoSe_2_-Gr even exhibited a superior HER catalytic performance, particularly when the HER was performed at a current density higher than ca. −55 mA·cm*^−^*^2^. Specifically, the MoSe_2_-Gr achieved −150 mA·cm*^−^*^2^ HER current density at an overpotential of 350 mV, while the benchmark Pt/C catalyst could achieve this current density only at a significantly higher HER overpotential of 591 mV (Figure 4a,b). 

The geometrical area-based current density discussed above in fact depends on the morphology of the deposited film in the given mass loading of the catalyst. Therefore, to examine the intrinsic catalytic activity of the catalysts toward HER, the mass activity-based LSV polarization curves of the MoSe_2_, MoSe_2_-Gr, and the benchmark Pt/C were measured and presented in Figure S2a. In addition, the corresponding mass activity profile at a given HER overpotential was also displayed in Figure S2b. In line with the geometrical area-based HER activity, the MoSe_2_-Gr catalyst exhibited significantly higher mass activity toward HER compared to that of the as-prepared pristine MoSe_2_ catalyst. Furthermore, the MoSe_2_-Gr catalyst demonstrated a comparable mass activity to that of the Pt/C at an overpotential of 250 mV. Beyond this, the MoSe_2_-Gr catalyst even exhibited superior mass activity toward the HER, indicating its excellent electrocatalytic performance. The Tafel slope, which is a fundamental parameter in evaluating the HER kinetics of electrocatalysts, was shown in Figure 4c. The corresponding Tafel slopes of MoSe_2_, MoSe_2_-Gr, and Pt/C in a 0.5 M H_2_SO_4_ electrolyte were determined to be 95, 67, and 51 mV.dec^−1^, respectively. A lower Tafel slope indicates that the HER took place at a faster rate on the surface of the MoSe_2_-Gr catalyst-based cathode compared to that of the MoSe_2_ catalyst. This finding suggests that the MoSe_2_-Gr catalyst had an enhanced HER kinetic, further supporting its superior electrocatalytic performance. The electrochemical impedance spectroscopy (EIS) of MoSe_2_ and MoSe_2_-Gr was applied to gain insights into their electrochemical kinetics. Figure 4d shows the Nyquist plots obtained from the EIS, which reveal that the charge transfer resistance for the HER process at the MoSe_2_-Gr/electrolyte interface is significantly lower compared to that at the MoSe_2_/electrolyte interface. This can be attributed to the expanded interlayer spacing of MoSe_2_ and the presence of graphene in the MoSe_2_-Gr catalyst. The hybrid structure of the MoSe_2_-Gr catalyst has, thus, provided a higher surface area and more active sites, facilitating the movement of electrons and ions between the catalyst and electrolyte. This may be the key reason for an improved HER performance of the MoSe_2_-Gr catalyst. Furthermore, the electrocatalytic HER performance of the MoSe_2_-Gr catalyst in 0.5 M H_2_SO_4_ is highly competitive with other MoSe_2_-based composites, as indicated in Table S1. This demonstrates the favorable performance and potential applicability of the MoSe_2_-Gr catalyst designed in this work for green hydron production via HER.

**Figure 5:**
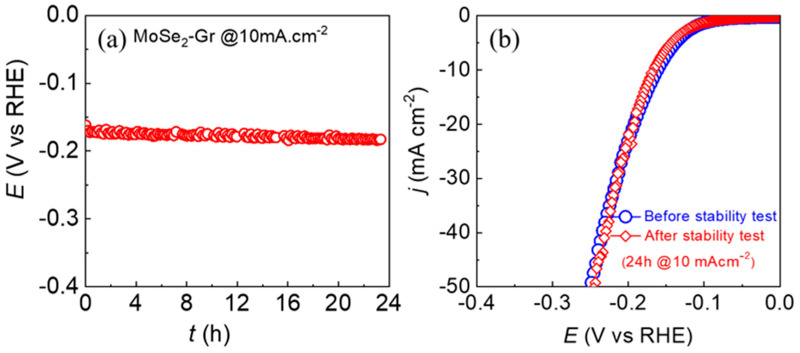
(**a**) Electrochemical stability test chronopotentiometric curve of MoSe_2_- Gr catalyst at an applied bias of −10 mAcm^−2^ in 0.5 M H_2_SO_4_, (**b**) the LSV curves of MoSe_2_- Gr against HER before and after 24 h durability test (**a**).

The MoSe_2_-Gr electrode was subjected to a chronopotentiometry test at a cathodic bias of −10 mA.cm^−2^ to evaluate its electrochemical stability. The resulting chronopotentiometry trace, as shown in Figure 5a, indicates that the MoSe_2_-Gr electrode exhibited excellent endurance over a period of 24 h. Furthermore, after the stability test for 24 h, a similar linear sweep voltammogram curve of the MoSe_2_-Gr catalyst to that of the one before the stability test was recorded, as displayed in Figure 5b. This finding confirms the long-term electrochemical stability of the MoSe_2_-Gr catalyst during HER in acidic electrolytes. The electrode maintained its catalytic activity and performance even after the extended operation, highlighting its potential for practical applications requiring sustained electrochemical performance.

## 4. Conclusions

In summary, a facile but novel two-step synthesis strategy involving the combination of solvothermal reaction and calcination of the product was successfully employed to fabricate the MoSe_2_-Gr composite. The resulting MoSe_2_-Gr composite exhibited highly competitive electrochemical catalytic performance for the hydrogen evolution reaction (HER) in an acidic medium. The designed MoSe_2_-Gr catalyst demonstrated a significantly improved catalytic activity compared to the as-prepared pristine MoSe_2_ with a lower Tafel slope of 67 mV dec^−1^ and a lower benchmark HER overpotential of 161 mV to drive the electrolysis at −10 mAcm^−2^. Remarkably, the MoSe_2_-Gr catalyst exhibited a closer HER catalytic activity to that of the state-of-the-art Pt/C catalyst at a catalytic current density of ca. −50 mA cm^−2^. Furthermore, extending the electrolysis for HER beyond ca. −55 mA cm^−2^, a superior HER catalytic activity than that of the benchmark Pt/C catalyst was achieved. The enhanced and superior HER performance of the MoSe_2_-Gr catalyst can be attributed to the in situ integrated graphene into the MoSe_2_ layers resulting in an interlayer expansion. This enhanced the surface area and concentration of HER active edge sites. Additionally, the larger interlayer spacing of the MoSe_2_-Gr composite might have facilitated the free movement of charges and ions between the MoSe_2_ interlayers and electrolytes, further enhancing the catalytic activity. Moreover, the MoSe_2_-Gr catalyst exhibited long-term electrochemical stability against HER in acidic electrolytes. These findings may open an avenue to the development of efficient and sustainable electrocatalysts for green energy conversion and storage.

## Figures and Tables

**Table 1:** Elements (N, C, H) analysis (weight percentage: wt%) of MoSe_2_ and MoSe_2_-Gr samples by the elemental analyzer.

Sample Name	Nitrogen	Carbon	Hydrogen
MoSe_2_	1.635	11.632	0.228
MoSe_2_-Gr	1.700	7.210	0.995

## Data Availability

The data presented in this study are available in this article and Supplementary Materials.

## Supplementary Materials

The following supporting information can be downloaded at: https://www.mdpi.com/article/10.3390/nano13142139/s1, Figure S1: BET surface area analysis results; Figure S2: Mass activity-based cathodic polarization curves and HER activity at difference overpotentials; Table S1: HER performance comparison of MoSe_2_ and their carbonous composites.
