# Supplementary material for: Escalating Catalytic Activity for Hydrogen Evolution Reaction on MoSe2@Graphene Functionalization
Source: Nanomaterials (Basel). 2023 Jul 23;13(14):2139. doi: 10.3390/nano13142139 (PMC10384179; doi:10.3390/nano13142139)
Supplement: Supplementary file 1 [file nanomaterials-13-02139-s001.zip › nanomaterials-2519075-supplementary.pdf]

Supporting information

Escalating catalytic activity for hydrogen evolution reaction on $MoSe_2$@graphene functionalization

Hoa Thi Bui [1*], Nguyen Duc Lam[1], Do Chi Linh[1], Nguyen Thi Mai[1], HyungIl Chang[2], Sung-Hwan Han[2], Vu Thi Kim Oanh[3], Anh Tuan Pham[4] , Supriya A. Patil[5], Nguyen Thanh Tùng[1*], Nabeen K. Shrestha[6*]

[1]Institute of Materials Science, Vietnam Academy of Science and Technology, Hanoi, Vietnam; hoabt@ims.vast.ac.vn (H.T.B); lamnd@ims.vast.ac.vn (N.D.L); linhdc@ims.vast.ac.vn (D.C.L); maint@ims.vast.ac.vn (N.T.M), tungnt@ims.vast.ac.vn (N.T.T)

[2]Department of Chemistry, Hanyang University, 222, Wangsimni-ro, Seongdong-gu, Seoul 04763, Republic of Korea; doctor99106@gmail.com (H.C); shhan@hanyang.ac.kr (S.H.H)

[3]Institute of Physic Physic and Graduate University of Science and Technology, Vietnam Academy of Science and Technology, Hanoi, Vietnam; oanhvtk@iop.vast.vn (V.T,K.O)

[4]Institute of Engineering and Technology, Thu Dau Mot University, Binh Duong, Vietnam; anhpt195@tdmu.edu.vn (A.T.P)

[5]Department of Nanotechnology and Advanced Materials Engineering, Sejong University, Seoul 05006, Republic of Korea; supriya2812@sejong.ac.kr (S.A.P.)

[6]Division of Physics and Semiconductor Science, Dongguk University, Seoul 04620, Republic of Korea;nabeenkshrestha@dongguk.ed (N.K.S)

*Correspondence:
Hoa Thi Bui (hoabt@ims.vast.ac.vn),
Nguyen Thanh Tung (tungnt@ims.vast.ac.vn),
Nabeen K. Shrestha (nabeenkshrestha@dongguk.ed).

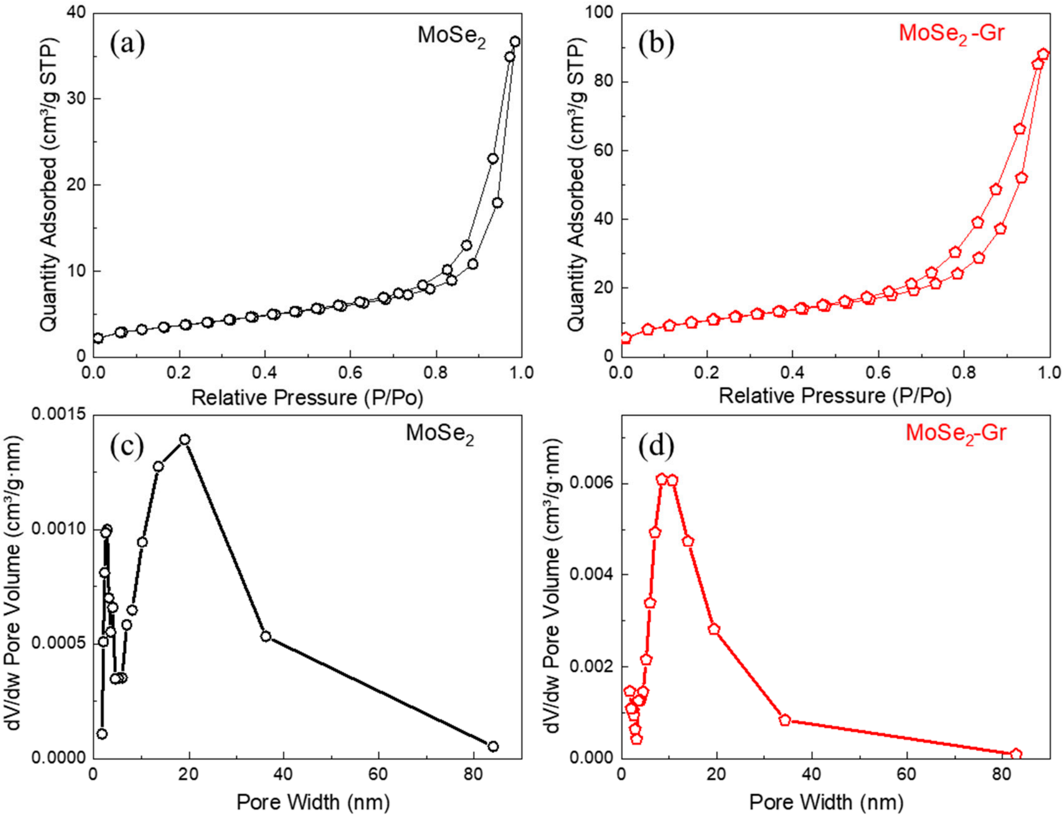

Figure S1. BET surface area analysis results: N_2-adsorption/desorption isotherms of (a) $MoSe_2$ and (b) $MoSe_2$-Gr. Pre size distribution of (c) $MoSe_2$ and (d) $MoSe_2$-Gr samples.

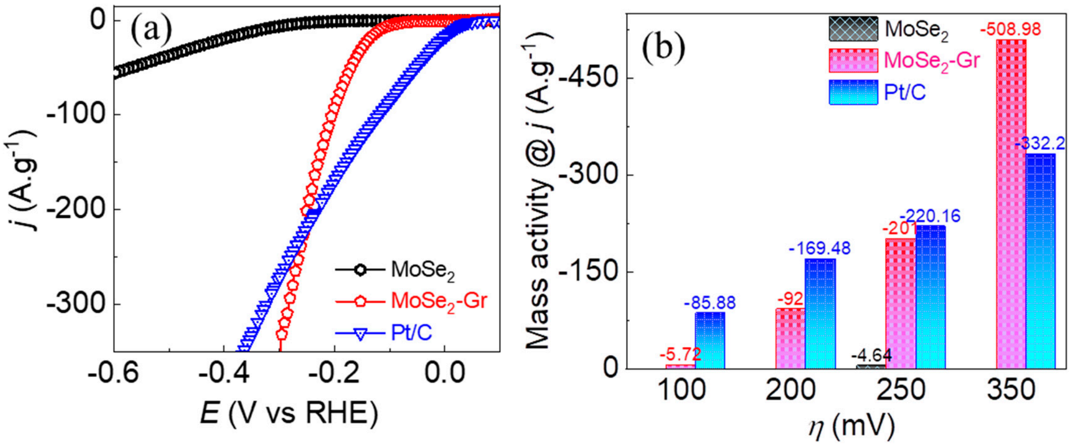

Figure S2. (a) Mass activity of $MoSe_2$, $MoSe_2$-Gr and Pt/C for HER performance in 0.5M H_2SO_4 solution. (b) Corressponding mass activity profile at various HER overpotentials.

Table S1. Electrocatalytic HER performance of $MoSe_2$ and their carbonous composites in 0.5 M H_2SO_4.

Catalyst	Mass loading ($mgcm^{-2}$)	Tafel slope (mV dec^{-1})	Overpotential @ 10 mA cm^{-2} (mV)	**References**
$MoSe_2$-Gr	0.25	67	161	This work
N-doped 1T-2H $MoSe_2$/G composites		67	153	[1]
C@$MoSe_2$ core-shell composites	0.285	72	270	[2]
double-shelled N-doped carbon@$MoSe_2$ hollow nanoboxes		55	102	[3]
defect-rich $MoSe_2$ with nitrogen-doped reduced		69	120	[4]

graphene oxide ($MoSe_2$/NG)				
$MoSe_2$/rGO-graphite-like membrane		51	150	[5]
$MoSe_2$/graphene nanosheet	0.7	61	159	[6]
$MoSe_2$/graphene hybrid	0.285	67	195	[7]
CNT@$MoSe_2$ hybrid	0.285	58	178	[8]
$MoSe_2$/Carbon Fiber	0.570	62	179	[9]
$MoSe_2$ Embedded CNT-rGO	-	53	240	[10]
$MoSe_2$–rGO	0.25	57	210	[11]

References

1. Huang, S.-Y.; Le, P.-A.; Nguyen, V.-T.; Lu, Y.-C.; Sung, C.-W.; Cheng, H.-W.; Hsiao, C.-Y.; Dang, V.D.; Chiu, P.-W.; Wei, K.-H. Surface plasma–induced tunable nitrogen doping through precursors provides 1T-2H MoSe2/graphene sheet composites as electrocatalysts for the hydrogen evolution reaction. *Electrochim. Acta* **2022**, *426*, 140767, doi.org/10.1016/j.electacta.2022.140767.

2. Ren, X.; Yao, Y.; Ren, P.; Wang, Y.; Peng, Y. Facile sol-gel synthesis of C@MoSe2 core-shell composites as advanced hydrogen evolution reaction catalyst. *Mater. Lett.* **2019**, *238*, 286–289, doi.org/10.1016/j.matlet.2018.12.036.

3. Chen, W.; Qiao, R.; Song, C.; Zhao, L.; Jiang, Z.-J.; Maiyalagan, T.; Jiang, Z. Tailoring the thickness of MoSe2 layer of the hierarchical double-shelled N-doped carbon@MoSe2 hollow nanoboxes for efficient and stable hydrogen evolution reaction. *J. Catal.* **2020**, *381*, 363–373, doi.org/10.1016/j.jcat.2019.11.013.

4. Zheng, D.; Cheng, P.; Yao, Q.; Fang, Y.; Yang, M.; Zhu, L.; Zhang, L. Excess Se-doped MoSe2 and nitrogen-doped reduced graphene oxide composite as electrocatalyst for hydrogen evolution and oxygen reduction reaction. *J. Alloys Compd.* **2020**, *848*, 156588, doi.org/10.1016/j.jallcom.2020.156588.

5. Poorahong, S.; Somnin, C.; Malam Mahamadou, I.; Dubois, C.; Chergui, S.; Peng, Z.; Su, Y.; Xuan Tran, T.; Thammakhet-Buranachai, C.; Mazzah, A.; et al. Nanoporous Graphite-like Membranes Decorated with MoSe2 Nanosheets for Hydrogen Evolution. *ACS Appl. Nano Mater.* **2022**, *5*, 2769–2778, doi.10.1021/acsanm.1c04431.

6. Mao, S.; Wen, Z.; Ci, S.; Guo, X.; Ostrikov, K. (Ken); Chen, J. Perpendicularly Oriented MoSe2/Graphene Nanosheets as Advanced Electrocatalysts for Hydrogen

Evolution. *Small* **2015**, *11*, 414–419, doi.org/10.1002/smll.201401598.

7. Liu, Z.; Li, N.; Zhao, H.; Du, Y. Colloidally synthesized MoSe2/graphene hybrid nanostructures as efficient electrocatalysts for hydrogen evolution. *J. Mater. Chem. A* **2015**, *3*, 19706–19710, doi.10.1039/c5ta05223h.

8. Huang, Y.; Lu, H.; Gu, H.; Fu, J.; Mo, S.; Wei, C.; Miao, Y.-E.; Liu, T. A CNT@MoSe2 hybrid catalyst for efficient and stable hydrogen evolution. *Nanoscale* **2015**, *7*, 18595–18602, doi.10.1039/C5NR05739F.

9. Zhang, Y.; Zuo, L.; Zhang, L.; Huang, Y.; Lu, H.; Fan, W.; Liu, T. Cotton Wool Derived Carbon Fiber Aerogel Supported Few-Layered MoSe2 Nanosheets As Efficient Electrocatalysts for Hydrogen Evolution. *ACS Appl. Mater. Interfaces* **2016**, *8*, 7077–7085, doi.10.1021/acsami.5b12772.

10. Park, G.D.; Kim, J.H.; Park, S.-K.; Kang, Y.C. MoSe2 Embedded CNT-Reduced Graphene Oxide Composite Microsphere with Superior Sodium Ion Storage and Electrocatalytic Hydrogen Evolution Performances. *ACS Appl. Mater. Interfaces* **2017**, *9*, 10673–10683, doi.10.1021/acsami.7b00147.

11. Park, S.-K.; Park, G.D.; Ko, D.; Kang, Y.C.; Piao, Y. Aerosol synthesis of molybdenum diselenide–reduced graphene oxide composite with empty nanovoids and enhanced hydrogen evolution reaction performances. *Chem. Eng. J.* **2017**, *315*, 355–363, doi.org/10.1016/j.cej.2017.01.032.
